# Hotspot of Crimean-Congo Hemorrhagic Fever Virus Seropositivity in Wildlife, Northeastern Spain

**DOI:** 10.3201/eid2709.211105

**Published:** 2021-09

**Authors:** Johan Espunyes, Oscar Cabezón, Lola Pailler-García, Andrea Dias-Alves, Lourdes Lobato-Bailón, Ignasi Marco, Maria P. Ribas, Pedro E. Encinosa-Guzmán, Marta Valldeperes, Sebastian Napp

**Affiliations:** Universitat Autònoma de Barcelona, Bellaterra, Spain (J. Espunyes, O. Cabezón, A. Dias-Alves, L. Lobato-Bailón, I. Marco, M.P. Ribas, P.E. Encinosa-Guzmán, M. Valldeperes);; Centre de Recerca en Sanitat Animal, Bellaterra (O. Cabezón, L. Pailler-García, S. Napp)

**Keywords:** Crimean-Congo hemorrhagic fever, CCHFV, CCHF, emerging infectious diseases, Ixodidae, tickborne disease, Spain, wild animals, zoonoses, viruses, vector-borne infections, ticks

## Abstract

We conducted a serosurvey for Crimean-Congo hemorrhagic fever virus antibodies in various wildlife species in Catalonia, northeastern Spain. We detected high seroprevalence in southern Catalonia, close to the Ebro Delta wetland, a key stopover for birds migrating from Africa. Our findings could indicate that competent virus vectors are present in the region.

Crimean-Congo hemorrhagic fever virus (CCHFV) is an arthropodborne *Orthonairovirus* mainly transmitted by ticks. In humans, CCHFV infection can cause severe and even fatal Crimean-Congo hemorrhagic fever (CCHF) disease (*1*). CCHFV also can infect wild and domestic mammalian species, producing viremia but causing a predominantly asymptomatic disease and such species have a role in the maintenance of the virus in the environment (*2*).

CCHFV is endemic in Africa, Asia, and eastern Europe but has more recently emerged in southwestern Europe. In 2010, CCHFV was detected in central-western Spain in *Hyalomma lusitanicum* ticks collected from red deer (*Cervus elaphus*) (*3*). In 2016, 2 autochthonous human CCHF cases were reported in Spain, 1 likely contracted through tick bite and the other caused by nosocomial transmission (*4*). Since then, 6 other CCHF clinical cases, including a retrospectively identified case from 2013, have been reported in the country, all of which are suspected to be caused by infected ticks (*5*,*6*). Further surveys on ticks (*7*,*8*), and serologic studies in humans (*9*) and animals (*10*) have shown evidence of CCHFV circulation in several areas of central and southwestern Spain. The high genetic variability of the CCHFV strains identified in Spain, including genotypes Africa III and IV and Europe V, are indicative of repeated introductions (*7*,*8*). The area of CCHFV detection coincides with the region where the ecologic conditions are more favorable for the presence of *H. marginatum* and *H. lusitanicum* ticks, the main vectors of the disease. Neither of these species have been reported in northeastern Spain, but ecologic models predict the existence of areas suitable for *H. marginatum* (*11*). To evaluate possible CCHFV circulation in Catalonia, northeastern Spain, we conducted a serosurvey to detect CCHFV antibodies in different susceptible wild animal species. 

## The Study

**Figure 1:**
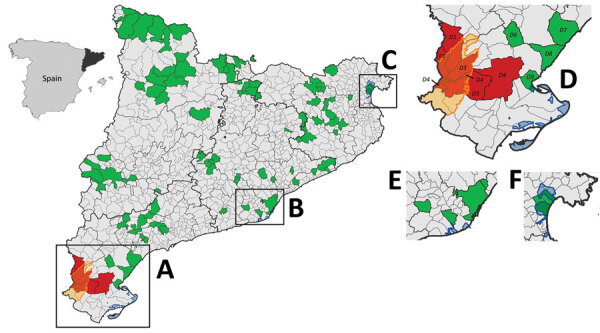
Distribution of areas sampled for detection of antibodies against Crimean-Congo hemorrhagic fever virus (CCHFV) in various species, Catalonia, northeastern Spain. Inset at left shows Catalonia (black) in northeastern Spain. Large map shows distribution of serosurveys throughout Catalonia: A) Ebro Delta; B) Llobregat Delta; C) Aiguamolls de l’Empordà; Enlarged areas represent regions with wetlands (blue shading), which are stopovers for migratory birds from Africa: D) Ebro Delta; E) Llobregat Delta; F) Aiguamolls de l’Empordà. Green shading indicates areas from which all samples were seronegative; red shading indicates >1 sample was seropositive; gray shading indicates area was not sampled; yellow shading/outline indicates location of Ports de Tortosa-Beseit National Park. Additional details are provided on CCHFV hotspots in Ebro Delta (D), which are close to and overlap wetlands and Ports de Tortosa-Beseit Natural Park. Among regions in this area, animals tested (no. positive/no. tested) included the following: D1, Iberian ibexes 10/10, wild boar 4/21; D2, Iberian ibexes 17/17, roe deer 1/1, wild boar 1/3; D3, Iberian ibexes 3/3; D4, Iberian ibexes 8/8, European rabbit 0/2; D5, Iberian ibexes 28/28, European rabbit 0/2; D6, European rabbit 0/6; D7, roe deer 0/1; D8, European rabbit 0/1; and D9, European rabbit 0/1.

**Figure 2:**
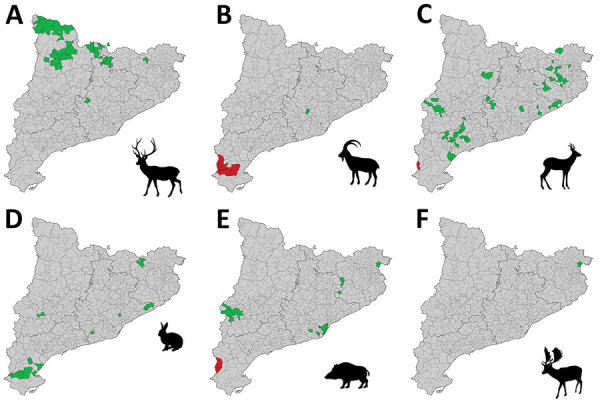
Distribution of areas sampled for detection of antibodies against Crimean-Congo hemorrhagic fever virus CCHFV by species, Catalonia, northeastern Spain. Green indicates all samples were seronegative; red indicates >1 sample was seropositive; gray indicates areas not sampled. A) Red deer (*Cervus elaphus*); B) Iberian ibex (*Capra pyrenaica*); C) roe deer (*Capreolus capreolus*); D) European rabbit (*Oryctolagus cuniculus*); E) wild boar (*Sus scrofa*); F) fallow deer (*Dama dama*).

Serum samples from different wildlife species were collected during 2014–2020 as part of routine wildlife surveillance in Catalonia from areas representing different ecosystems (Figures 1, 2). We tested for CCHFV antibodies in serum samples from 174 red deer, 84 Iberian ibexes (*Capra pyrenaica*), 79 roe deer (*Capreolus capreolus*), 35 European rabbits (*Oryctolagus cuniculus*), 156 wild boars (*Sus scrofa*), and 4 fallow deer (*Dama dama*) (Table 1). We used the CCHF Double Antigen Multi-species ELISA kit (IDvet, https://www.id-vet.com), which has a sensitivity of 98.9% (95% CI 96.8%–99.8%) and a specificity of 100% (95% CI 99.8%–100%) (*12*). 

**Table 1:** Distribution of samples tested for the presence of antibodies against CCHFV among various mammalian species, Catalonia, Spain*

Species	2014–2016	2017	2018	2019	2020	Total
Red deer	0/13 (0%–28%)	0/60 (0%–1%)	0/15 (0%–3%)	0/29 (0%–15%)	0/57 (0%–8%)	0/174 (0%–3%)
Iberian ibex		15/15 (75%–100%)	5/5	46/46 (90%–100%)	0/18 (0%–22%)	66/84 (68%–87%)
Roe deer		0/1	0/1	1/59 (0%–10%)	0/18 (0%–22%)	1/79 (0%–8%)
European rabbit	0/21 (0%–19%)	0/11 (0%–32%)	0/3			0/35 (0%–12%)
Wild boar		1/87 (0%–7%)	3/3		1/48 (0%–13%)	5/156 (1%–8%)
Fallow deer				0/4		0/4
Total	0/34 (0%–13%)	16/174 (6%–15%)	8/27 (15%–50%)	47/156 (23%–38%)	1/141 (0%–5%)	72/532 (11%–17%)

*Data are no. positive/no. tested (95% CI for percentage CCHFV positive). CCHFV, Crimean-Congo hemorrhagic fever virus.

Because CCHFV might have been introduced in the region via ticks carried by migratory birds (*3*), we selected 226 samples from areas close to the 3 main points of arrival of birds from Africa: the wetlands of the Ebro Delta (n = 101); the Llobregat Delta (n = 82), in close proximity to the urban area of Barcelona; and the Aiguamolls de l’Empordà (n = 43). The remaining 306 samples were collected from municipalities throughout Catalonia.

Of 532 samples tested, CCHFV antibodies were detected in 72 animals, including Iberian ibex (66/84), roe deer (1/79), and wild boar (5/156) (Tables 1, 2). All 72 seropositive samples came from the same area in southern Catalonia, which includes 5 municipalities within or close to the Ports de Tortosa-Beseit Natural Park (Figure 1). This area is composed of rugged terrain, including canyons and ravines, and mainly is covered by a Mediterranean forest dominated by oaks, pines, and dense shrubland. This natural area is located a few kilometers from the Ebro Delta, one of the main wetlands in Spain and a key stopover for birds migrating from Africa to Europe. Thus, CCHFV introduction via infected ticks transported by migrating birds seems plausible.

**Table 2:** Distribution of samples tested for the presence of antibodies against CCHFV among various mammalian species, Ebro Delta area, Spain*

Species	2014–2016	2017	2018	2019	2020	Total
Iberian ibex		15/15 (75%–100%)	5/5	46/46 (90%–100%)		66/66 (93%–100%)
Roe deer				1/2		1/2
European rabbit	0/11 (0%–32%)					0/11 (0%–32%)
Wild boar		1/1	3/3	0/18 (0%–22%)	1/2	5/24 (8%–43%)
Total	0/11 (0%–32%)	16/16 (76%–100%)	8/8 (60%–100%)	47/66 (59%–81%)	1/2	72/103 (60%–78%)

*Data are no. positive/no. tested (95% CI for percentage CCHFV positive). CCHFV, Crimean-Congo hemorrhagic fever virus.

The 66 Iberian ibexes tested in the affected area during 2017–2019, and 1/2 roe deer sampled in 2019, were CCHFV-positive, indicating high seroprevalence in the area since at least 2017. A 2018 serosurvey in wild ruminants also found a high seroprevalence (79%) in some areas of central Spain known to have *Hyalomma* ticks but where CCHFV had not been detected previously (*10*). In contrast, of 24 wild boars sampled from affected municipalities during 2017–2020, only 5 (20.8%) were seropositive. Reasons for the difference in seroprevalence between Iberian ibexes and wild boars are not clear and will require additional studies. One possible explanation would be that adult *Hyalomma* ticks feed preferentially on the family Bovidae (*13*); high seroprevalences frequently are observed in Spain among domestic goats (*Capra aegagrus hircus*), a closely related species (*10*). European rabbits tested in the affected area were seronegative (Table 2); however, they were sampled in 2016 when CCHFV might not have been introduced or might have been at lower levels. No CCHFV antibodies were detected in red deer or fallow deer, but in the areas where they were sampled, seropositivity was not detected in any of the other susceptible species either (Figure 2). 

## Conclusions

Detection of CCHFV antibodies among animals in southern Catalonia implies the availability of competent vectors, most likely *H. marginatum* ticks; however, presence of *H. marginatum* ticks in the area and on the host species will need to be confirmed. The range of *H. marginatum* ticks is expanding in Europe; permanent populations have been reported in southern France (*14*). This expansion probably is influenced by the density of wild ungulates, from which adult *H. marginatum* ticks feed, and leporids, from which immature ticks feed. In Catalonia, increasing populations of rabbits and wild ungulates, including wild boar, roe deer, and fallow deer, have required management measures to control their populations in recent years (*15*). 

Besides southern Catalonia, samples from other areas evaluated in this study were seronegative. Whether seronegativity results from the absence of competent vectors or the absence of CCHFV is unclear, but defining seronegative and seropositive areas will be key in assessing risk for CCHFV transmission in the Mediterranean ecologic region. Further serosurveys to identify amplifying hosts and reservoirs of CCHFV in this ecologic region could help determine whether additional prevention measures against zoonotic transmission are needed in the area. Moreover, detecting the virus in hosts or vectors from the affected area and phylogenetic studies could clarify the origin of CCHFV in Catalonia. Risk for further introduction of CCHFV via migratory birds or expansion from the currently affected area to unaffected areas underscore the need for continued CCHF disease surveillance in Catalonia. 
